# Molecular Skin Surface-Based Transformation Visualization between Biological Macromolecules

**DOI:** 10.1155/2017/4818604

**Published:** 2017-04-20

**Authors:** Ke Yan, Bing Wang, Holun Cheng, Zhiwei Ji, Jing Huang, Zhigang Gao

**Affiliations:** ^1^College of Information Engineering, China Jiliang University, 258 Xueyuan Street, Hangzhou 310018, China; ^2^The Advanced Research Institute of Intelligent Sensing Network, Tongji University, 4800 Caoan Road, Shanghai 201804, China; ^3^School of Computing, National University of Singapore, 21 Lower Kent Ridge Road, Singapore 119077; ^4^School of Information & Electronic Engineering, Zhejiang Gongshang University, 18 Xuezheng Road, Hangzhou 310018, China; ^5^CNRS LTCI, Telecom ParisTech, 46 rue Barrault, 75013 Paris, France; ^6^College of Computer Science, Hangzhou Dianzi University, Hangzhou 310018, China

## Abstract

Molecular skin surface (MSS), proposed by Edelsbrunner, is a *C*^2^ continuous smooth surface modeling approach of biological macromolecules. Compared to the traditional methods of molecular surface representations (e.g., the solvent exclusive surface), MSS has distinctive advantages including having no self-intersection and being decomposable and transformable. For further promoting MSS to the field of bioinformatics, transformation between different MSS representations mimicking the macromolecular dynamics is demanded. The transformation process helps biologists understand the macromolecular dynamics processes visually in the atomic level, which is important in studying the protein structures and binding sites for optimizing drug design. However, modeling the transformation between different MSSs suffers from high computational cost while the traditional approaches reconstruct every intermediate MSS from respective intermediate union of balls. In this study, we propose a novel computational framework named general MSS transformation framework (GMSSTF) between two MSSs without the assistance of union of balls. To evaluate the effectiveness of GMSSTF, we applied it on a popular public database PDB (Protein Data Bank) and compared the existing MSS algorithms with and without GMSSTF. The simulation results show that the proposed GMSSTF effectively improves the computational efficiency and is potentially useful for macromolecular dynamic simulations.

## 1. Introduction

Physically understanding the protein-protein or protein-RNA interactions involved in the biological processes is essential for new drug design in biomedical research [1, 2]. Biological macromolecular surface visualization in 3D space helps us to focus on the spatial and structural information of proteins at atomic level and further explains the dynamic changes of molecular structure timely. Biological macromolecule (such as proteins) surface representation has been an important subject in the field of molecular visualization since 1964 [3], where atoms are represented by 3-dimensional spherical structures with the sphere centers at actual atom positions and radii representing the electron fields. For example, the public database PDB (Protein Data Bank) [4] provides the spatial information about the amino acids in each protein in 3D space. The studies of macromolecule surface visualization provide an effective way to improve our understanding of the complex biological processes and functions in tumor cells [5]. In clinic, the aim of modeling the protein surface representation is to potentially reveal the key domains involved in protein-related interactions for optimizing drug design in biotechnology and food industry [6–9].

**Figure 1:**
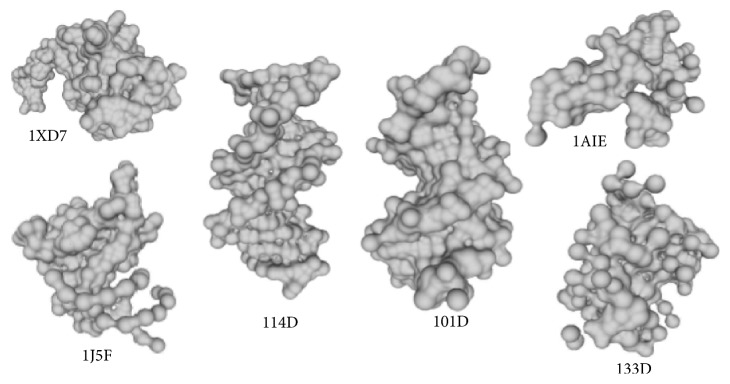
Examples of the molecular skin surfaces with PDB numbers: 1XD7, 1J5F, 114D, 101D, 1AIE, and 133D.

In 1999, Edelsbrunner introduced a new paradigm of molecular surface class called the molecular skin surface (MSS) [10]. The MSS is a smooth transformable surface which was soon utilized in the macromolecular visualization assisting drug design and biomedical research [11]. Compared to traditional molecular surface representations, including Van der Waals surface [12], SAS [13], and SES [14], the MSS has the advantages of easy construction, no self-intersection, *C*^2^ continuous surface, being decomposable to quadratic patches, and being transformable between multiple MSSs. The static construction of MSS is simple and straightforward by considering all atoms as “balls” in ℝ^3^. A special complex named “mixed cells” is constructed by taking the Minkowski sum of Delaunay and Voronoi complexes constructed by all balls. In each mixed cell, the MSS patch is either a sphere patch or a hyperboloid patch, which can be constructed by triangular meshes, or directly rasterized by ray casting.  Figure 1 shows several examples of the MSS.

**Figure 2:**
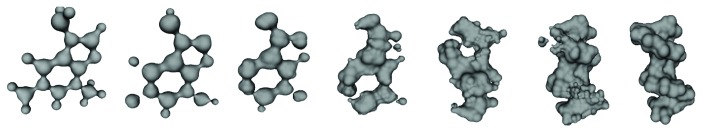
Transformation from a caffeine MSS to a protein MSS.

The MSS transformation shows an animation process between two MSSs, namely, source MSS and target MSS. For example, Figure 2 shows a dynamic transformation process between a caffeine molecule and a protein; both molecules are modeled with PDB files. Such transformation visualization processes have decent potentials, for example, visualizing the processes of macromolecular dynamics to assist drug design and bioinformatics research [15–18]. However, existing static MSS algorithms slice the animation into a number of time frames and reconstruct each intermediate MSS from the union of balls to achieve the goal. At each step, the underlying Delaunay and Voronoi complexes and mixed cells are reconstructed on CPU. The computational complexity hinders the MSS to be widely used in modern molecular visualization softwares. Moreover, there are concerns about the animation discontinuity because of the discrete slicing and no surface point correspondence between the previous time frame and the next time frame.

In this study, we propose a general MSS transformation framework (GMSSTF) without reconstructing the intermediate MSS from unions of balls [19, 20]. First, by taking two input unions of balls and constructing the corresponding Delaunay and Voronoi complexes and mixed cells, we demonstrate a continuous transformation from the source mixed cell to the target mixed cell. Second, with the continuous mixed cell centers and various weights, we build the MSS in the next time frame by translation and scaling from the current frame. Third, we apply the GMSSTF to partial molecular movements, which is potentially useful in mimicking a single molecule movements. Last, we apply our algorithm to both CPU and GPU approaches of MSS construction to show the efficiency improvements. All experiments are performed with molecular structure downloaded from the public protein database PDB (Protein Data Bank http://www.rcsb.org/pdb/). In summary, the MSSTF has the following four contributions to the literature:

1. *Modeling Dynamic Animation Process*. It is the first computational framework which requires no reconstruction for the intermediate MSSs using unions of balls. Both the underlying mixed cells structure and the MSS are moving continuously with formulated mathematical expressions. The proposed framework therefore is able to generate high-quality animation process for the analyses in bioinformatics.
2. *Handling Partial Molecular Movements*. We demonstrate the usefulness of the proposed approach to the partial molecular movements, where the source and target molecules belong to the same molecule with different states (e.g., a protein in folding process). Some atoms positions are identical from the source to the target, which creates degenerate cases for *general position assumption.* We show the solution of handling such cases.
3. *Improving Computational Efficiency*. All intermediate mixed cells and MSSs are generated directly by moving the corresponding mixed cell elements or surface points from the previous time frame. The abovementioned approach saves the time of reconstructing the Delaunay and Voronoi complexes for intermediate MSSs. Experimental results show that the efficiency is largely improved compared to traditional static skin approaches.
4. *Recording Surface Point Correspondence*. Since each intermediate MSS is generated from the previous time frame by moving the surface points, there are surface point correspondences between neighboring frames. The surface point correspondence establishes an atom-atom linkage between the source, intermediate, and target molecules and can be an important evidence in macromolecular dynamics research.

In summary, the proposed computational framework of macromolecule MSS visualization improves the available techniques in the literature and can be potentially useful for protein interaction prediction and drug design.

## 2. Related Work

### 2.1. The Definition of MSS

The mathematical expression of a weighted point in ℝ^3^ is *b*_*i*_ = (*z*_*i*_, *w*_*i*_)  ∈  ℝ^3^ × ℝ, where *z*_*i*_  ∈  ℝ^3^ is the weighted point center and *w*_*i*_  ∈  ℝ is the weight. The definition of weighted point includes spheres; and the weighted point *b*_*i*_ can be also viewed as a sphere with center *z*_*i*_ and radius $\left(\left|w_{i}\right|/w_{i}\right)\sqrt{\left|w_{i}\right|}$. In addition, the weighted point allows the weight *w*_*i*_ to be negative. The weighted distance of a space point *x*  ∈  ℝ^3^ from a weighted point *b*_*i*_ is defined as *π*_*b*_*i*__(*x*) = ‖*xz*_*i*_‖^2^ − *w*_*i*_.

A MSS is constructed by a set of weighted points *B* = {*b*_*i*_  ∈  ℝ^3^ × ℝ | *i* = 1,  2,   …,  *n*}. Three weighted point operations are defined, including *addition*, *scalar multiplication*, and *square root* for *b*_*i*_,  *b*_*j*_  ∈  *B* and *γ*  ∈  ℝ, respectively:

(1) $$\begin{array}{c} b_{i}+b_{j}=\left(z_{i}+z_{j},w_{i}+w_{j}+2\langle z_{i},z_{j}\rangle \right), \end{array}$$

(2) $$\begin{array}{c} \gamma b_{i}=\left(\gamma z_{i},\gamma w_{i}+\left(\gamma^{2}-\gamma \right){\left\|z_{i}\right\|}^{2}\right), \end{array}$$

(3) $$\begin{array}{c} \sqrt{b_{i}}=\left(z_{i},w_{i}\right), \end{array}$$

where 〈*z*_*i*_, *z*_*j*_〉 is the dot product of *z*_*i*_ and *z*_*j*_.

The *convex hull* of *B* is defined as

(4) $$\begin{array}{c} \mathrm{conv}\left(B\right)=\left\{\sum \lambda_{i}b_{i}\left|\sum \lambda_{i}=1\right.,\;\lambda_{i}\geq 0,\;i=1,\;\ldots ,\;n\right\}. \end{array}$$

The MSS body is the union of all shrunken weighted points in conv(*B*), and the *C*^2^ continuous surface MSS is defined as the boundary of the union of all shrunken weighted points in conv(*B*), which is formally expressed as

(5) $$\begin{array}{c} \mathrm{skin}\left(B\right)=\partial \left(⋃\sqrt{\mathrm{conv}\left(B\right)}\right). \end{array}$$

**Figure 3:**
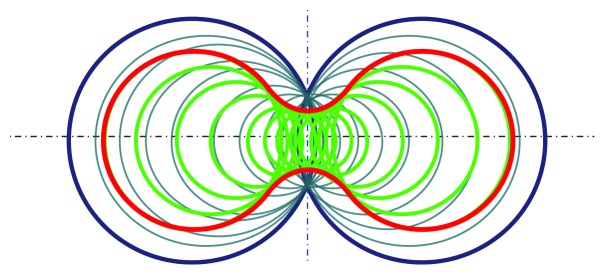
The red line shows the MSS defined by two weighted points marked in thick blue (*B*). All green circles represent $\sqrt{\text{conv}\left(B\right)}$.

Figure 3 depicts a MSS defined by two weighted points in ℝ^2^. The input weighted points are the two largest blue circles; and the MSS is drawn in red.

### 2.2. The Construction of MSS

**Figure 4:**
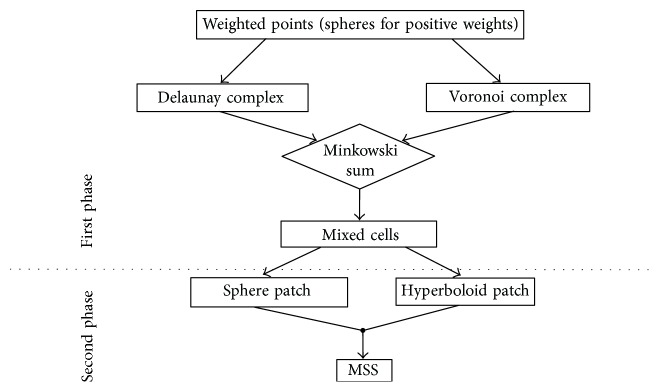
The flowchart of constructing a MSS in ℝ^3^.

In ℝ^3^, the MSS is constructed by a set of spheres with the sphere algebra. The resulting MSS is a *C*^2^ continuous surface defined by the convex hull of a set of shrunken spheres in ℝ^3^, which can be decomposed into sphere and hyperboloid patches using the definition of Delaunay and Voronoi complexes. The flowchart of the MSS construction process is shown in Figure 4. The whole process of MSS construction can be partitioned into two phases. For the first phase, we construct the Delaunay and Voronoi complexes from a union of balls/atoms and take the Minkowski sum to form a more complex structure called the mixed cells. The mixed cell structure partitions the MSS into patches. In each mixed cell, the MSS patch is either a formulated sphere patch or a formulated hyperboloid patch. In the second phase, we model and build every patch within its local mixed cell. The combination of all patches is the final MSS.

#### 2.2.1. The Delaunay and Voronoi Complexes and Mixed Cells

From the geometry insight, the Delaunay and Voronoi complexes can be easily obtained from the weighted point set *B* [21]. By basic definition, the *Voronoi region v*_*i*_ of *b*_*i*_  ∈  *B* can be defined by the equation:

(6) $$\begin{array}{c} v_{i}=\left\{x\;\in \;{\mathbb{R}}^{d}\left|\pi_{b_{i}}\left(x\right)\leq \pi_{b_{j}}\left(x\right),\;b_{j}\;\in \;B\right.\right\}, \end{array}$$

where *π*_*b*_*i*__(*x*) denotes the weighted distance from *x* to *b*_*i*_.

Then, a *Voronoi cell* of a set of weighted points *X* ⊆ *B* can be written as *v*_*X*_ = ⋂_*b*_*i*_∈*X*_*v*_*i*_, and the *Voronoi complex* of *B* is the collection of all *v*_*X*_ for *X* ⊆ *B* [22]. A *Delaunay cellδ*_*X*_ is a complementary geometric element of the Voronoi cell *v*_*X*_ [23]. The collection of all Delaunay cells is called the *Delaunay complex* of *B* and denoted as *D*_*B*_. The general position assumption is usually made so that the resulting Delaunay complex is always a simplicial complex. The *traditional general position (TGP) assumption* is defined as ∀*v*_*X*_  ∈  *V*_*B*_,  card(*X*) = dim(*δ*_*X*_) + 1 [24]. Under the TGP assumption, the Delaunay cells in ℝ^3^ can be summarized involving four types, namely, vertices, edges, triangles, and tetrahedra.

The mixed cell structure is a mixture of Delaunay and Voronoi complexes [10]. A mixed cell *μ*_*X*_ is formed by taking the Minkowski sum of *δ*_*X*_ and *v*_*X*_: *μ*_*X*_ = (*δ*_*X*_ + *v*_*X*_)/2. We also provide the definitions for the mixed cell center *z*_*X*_ and mixed cell weight *w*_*X*_:

(7) $$\begin{array}{c} z_{X}=\mathrm{aff}\left(\delta_{X}\right)\cap \mathrm{aff}\left(v_{X}\right), \end{array}$$

(8) $$\begin{array}{c} w_{X}=w_{i}-{\left\|z_{X}z_{i}\right\|}^{2}, \end{array}$$

where (*z*_*i*_, *w*_*i*_) is a ball orthogonal to *X* [21].

#### 2.2.2. Sphere and Hyperboloid Patches

The mixed cells partition the MSS into sphere and hyperboloid patches which both can be represented by quadratic equations in the standard forms:

(9) $$\begin{array}{c} x_{1}^{2}+x_{2}^{2}+x_{3}^{2}=R^{2}, \end{array}$$

(10) $$\begin{array}{c} x_{1}^{2}+x_{2}^{2}-x_{3}^{2}=\pm R^{2}, \end{array}$$

by translating *z*_*X*_ to the origin and orienting the symmetry axis to the *x*_3_-axis for hyperboloid patch cases. In (10), the ± sign represents the choices of one-sheet or two-sheet hyperboloid. The corresponding patch type of each mixed cell type is shown in Table 1. The table will be expanded while the TGP assumption is extended in superimposed Voronoi complexes [25].

#### 2.2.3. CPU and GPU Implementation of MSS Visualization

Since the MSS was born in 1999, in the last decade, many efforts have been made to fast visualize the MSS with guaranteed resolution in order for MSS to be used in biomedical science and engineering. On the CPU side, Kruithof and Vegter proposed to triangulate the MSS within each patch [26]. Since the overall MSS can be decomposed to quadratic patches, it is convenient for them to project each patch into 2 dimensions and triangulate the patch with small triangles. The problem of Kruithof and Vegter's approach is that the triangles have completely no quality control and will be possibly distorted during the process of projecting. In 2004 and 2005, Cheng and Shi improved the triangulation quality of MSS by introducing the restricted Delaunay triangulation to the mesh representation [27, 28]. Instead of meshing sampled points on the skin surface and controlling the triangle quality by edge contraction and circumcenter insertion, they triangulate the sample points in 3 dimensions and take the surface triangle as the final mesh. The 3-dimensional triangulation satisfies the Delaunay property.

On the GPU side, Chavent et al. [29, 30] utilized GPU to speed up the rasterization and promote the MSS to real-world molecular visualization in the field of bioinformatics. It is noted that although Chavent et al.'s approach is named as the GPU approach, they utilize the CPU construction of Delaunay and Voronoi complexes and mixed cells in the first phase. And the GPU processing based on rasterization using ray tracing is only applied in the second phase. Lindow et al. [31] demonstrated how to further accelerate Chavent's method by paralleling the first phase.

### 2.3. Existing Transformation Algorithms

Existing automatic transformation/morphing algorithms can be categorized into three types according to the surface representation of the source and target shapes. *Explicit surface transformation methods* [32, 33] represent objects in mesh form. The morphing trajectory is found by the vertices correspondence between the source and target shapes. By prespecified vertex correspondence or skeleton information, the explicit surface morphing methods handle morphings well between similar objects [34, 35]. However, it is usually difficult to handle topology changes automatically, for example, controlling splitting/merging and creation of holes and tunnels [36, 37]. *Volumetric transformation methods* [38] improve the explicit surface morphing methods by representing objects in voxels form. The transformation animation is achieved by mapping from one voxel in source shape to another voxel in target shape. One recent work by Wu et al. [39] shows that the volumetric transformation methods handle topology changes well. *Implicit surface transformation methods* [40–42] represent source and target shapes by implicit functions. Topology changes again can be well handled. However, both volumetric transformation methods and implicit surface transformation methods require user-defined information, such as representative vertex correspondence or skeletons of the source/target shapes.

To our knowledge, there is no transformation/morphing method available that satisfies all the following three criteria:

1. Handling topology changes automatically
2. Not requiring user-specified anchor points between source and target shapes
3. Not requiring skeleton information of source and target shapes.

The general molecular skin surface transformation framework (GMSSTF) that we proposed in this paper satisfies all the three requirements as stated above, which is potentially useful in mimicking macromolecular transformation/interactions in macromolecular dynamics studies.

## 3. Transformation between Different MSSs

In this section, we present a transformation/morphing framework between two MSSs. The proposing transformation framework translates, scales the intermediate mixed cell, and generates each intermediate MSS from its previous time frame, which has the potential to largely improve the computational efficiency of the transformation, regardless of the MSS representation. Moreover, the proposed method requires no external information such as anchor point assignments or skeleton information. The implicit function property of the MSSs guarantees the automatic topology change handling during the transformation process.

### 3.1. General MSS Transformation Framework

The general MSS transformation framework (GMSSTF) slowly transforms one MSS (source) to another MSS (target). The two MSSs can be the same molecule in different forms (Section 4), or two different molecules. The underlying Voronoi complex is mathematically proved as a fixed underlying structure by Chen and Cheng in 2006 [25]. The corresponding Delaunay complex transforms along with the intermediate MSS with linear interpolation.

The GMSSTF describes the vertex flow trajectories between two MSS triangular meshes without any extra information, such as skeleton or vertex correspondence. Suppose the source MSS mesh is positioned at time *t* = 0; and the target MSS mesh is positioned at time *t* = 1. All intermediate MSS meshes are generated by interpolation between the source shape and target shape with a *t*  ∈  (0,  1). While *B*_0_ and *B*_1_ are the weighted point sets of the source and target MSSs, the intermediate weighted point set *B*(*t*) is

(11) $$\begin{array}{c} B\left(t\right)=\left\{b_{ij}\left(t\right)=\left(1-t\right)b_{i}+{tb}_{j}\;\left|\;b_{i}\;\in \;B_{0},b_{j}\;\in \;\right.B_{1}\right\},\;for\;t\;\in \;\left[0,1\right], \end{array}$$

where *t*  ∈  (0, 1); and the intermediate MSS can be obtained directly from *B*(*t*) [43].

### 3.2. Intermediate Voronoi Complexes

The GMSSTF guarantees all intermediate MSSs share the same Voronoi complex structure, which is superimposition of the source Voronoi complex and the target Voronoi complex [25]:

(12) $$\begin{array}{c} V=\left\{v_{XY}\left|v_{X}\right.\;\in \;V_{source},\;v_{Y}\;\in \;V_{target},\;v_{X}\;\cap \;v_{Y}\neq \emptyset \right\}, \end{array}$$

where *V*_source_ and *V*_target_ are the source and target Voronoi complexes, respectively, constructed under TGP assumption.

For the superimposed Voronoi complex *V*, the TGP is violated, where we allow

(13) $$\begin{array}{c} \mathrm{dimension}\left(v_{XY}\right)=\mathrm{dimension}\left(v_{X}\right)+\mathrm{dimension}\left(v_{Y}\right)-d, \end{array}$$

where *d* is the Euclidean space dimension.

**Figure 5:**
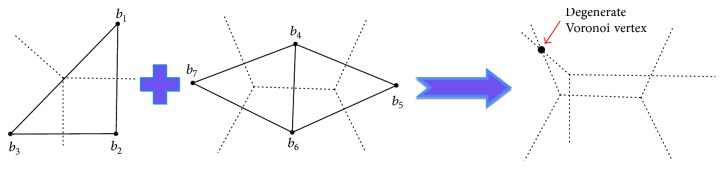
Superimposition of two Voronoi complexes constructed by two weighted point sets under SIGP assumption. The resulting intermediate Voronoi complex (right-most) has degenerate Voronoi cells such as the marked Voronoi vertex which is surrounded by four Voronoi regions.

Figure 5 illustrated a new type Voronoi vertex which is created by intersecting two Voronoi edges and bounded by four Voronoi regions. The new definition of general position assumption is named as superimposition general position (SIGP) assumption [25].

There are six possible intermediate Voronoi cell types under SIGP assumption after superimposing Voronoi complexes in ℝ^3^ (Table 2). Each type of intermediate Voronoi cells can be identified by a tuple: (dim(*v*_*X*_),  dim(*v*_*Y*_),  dim(*v*_*XY*_)), with the assumption that dim(*v*_*X*_) > dim(*v*_*Y*_). The first four Voronoi cell types in Table 2 are identical to the four Voronoi cell types in Table 1. The new types are the last two types, namely, (2,2,1) and (2,1,0), which represent the two situations violating the TGP assumption. Tuple (2,1,0) describes the case while a Voronoi face (shared by two Voronoi regions) intersects a Voronoi edge (shared by three Voronoi regions) at a vertex. This resulting Voronoi vertex is shared by six Voronoi regions under the SIGP assumption.

### 3.3. Transforming Delaunay Complexes and Intermediate Mixed Cells

We denote the intermediate Delaunay complex, *D*(*t*), as the Delaunay complex of *B*(*t*), and it is not a simplicial complex. Apart from regular Delaunay triangulation, we define the intermediate Delaunay complex as

(14) $$\begin{array}{c} D\left(t\right)=\left\{\mathrm{conv}\left(z\left(v^{-1}\left(v_{XY}\right)\right)\right)\left|v_{XY}\right.\;\in \;V\left(t\right)\right\}, \end{array}$$

where

(15) $$\begin{array}{c} z\left(X\right)=\left\{z_{i}\left|b_{i}\;\in \;X\right.\right\}, \end{array}$$

(16) $$\begin{array}{c} v^{-1}\left(v_{XY}\right)=\left\{b_{ij}\left(t\right)\left|v_{X}\in V_{0},v_{Y}\in V_{1},v_{X}\;\cap \;v_{Y}\neq \emptyset \right.\right\}. \end{array}$$

**Figure 6:**
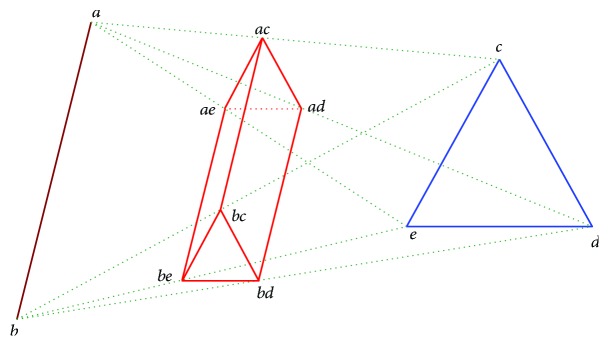
The linear interpolation between source vertices (*a*, *b*) and target vertices (*c*, *d*, and *e*) produces a transforming Delaunay cell which is a triangular prism.

With the fixed underlying intermediate Voronoi complex, every Delaunay cell in Table 2 can be translated and scaled according to linear interpolation between the source weighted point positions and target weighted point positions.  Figure 6 illustrates the linear interpolation from an edge of the source Delaunay complex and a triangle of the target Delaunay complex, which is the case of Tuple (2,1,0) in Table 2. All six vertices of the intermediate Delaunay triangular prism follow the linear interpolation from the source vertices to the target vertices.

Each intermediate mixed cell *μ*_*X*_(*t*) is calculated by taking the Minkowski sum of the corresponding Delaunay cell *δ*_*X*_(*t*) and Voronoi cell *v*_*X*_(*t*) (Table 2), where the mixed cell center *z*_*X*_(*t*) is determined by the intersection between Delaunay and Voronoi cells and the mixed cell weight *w*_*X*_(*t*) is calculated according to the mixed cell center position. The combination of the mixed cell center position and weight determines the skin patch within the mixed cell.

Since the intermediate Voronoi cells are fixed, and the intermediate Delaunay cells transform according to linear interpolation, the intermediate mixed cell center positions can be calculated by taking the linear interpolation between the source mixed cell centers and target mixed cell centers:

(17) $$\begin{array}{c} z_{X}\left(t\right)=\left(1-t\right)\cdot z_{X}\left(0\right)+t\cdot z_{X}\left(1\right), \end{array}$$

and the mixed cell weight is computed accordingly:

(18) $$\begin{array}{c} w_{X}\left(t\right)=w_{i}\left(t\right)-{\left\|z_{i}\left(t\right)z_{X}\left(t\right)\right\|}^{2}. \end{array}$$

### 3.4. Transforming Sphere and Hyperboloid Patches

The transformation of the MSS patches (either sphere or hyperboloid patches) relies on the transformation of the mixed cells. In each local coordinate system of an intermediate mixed cell *μ*_*X*_(*t*), the translation of the mixed cell center and the variance of the mixed cell weight determine the surface point moving trajectory of the intermediate MSS. Suppose a MSS surface point *p* at time *t*_0_ is known. The point position *p*(*t*_1_) at the next time frame can be calculated with the local mixed cell center translation *z*(*t*_1_) − *z*_*X*_(*t*_0_) and the mixed cell weight scaling factor $\sqrt{w\left(t_{1}\right)/w\left(t_{0}\right)}$:

(19) $$\begin{array}{c} p\left(t_{1}\right)=z\left(t_{1}\right)+\left(p\left(t_{0}\right)-z_{X}\left(t_{0}\right)\right)\cdot \sqrt{\frac{w\left(t_{1}\right)}{w\left(t_{0}\right)}}. \end{array}$$

The local mixed cell weight *w*(*t*) for both *t*_0_ and *t*_1_ can be calculated with the assistance of (17) and (18).

## 4. Partial Molecular Movements

**Figure 7:**
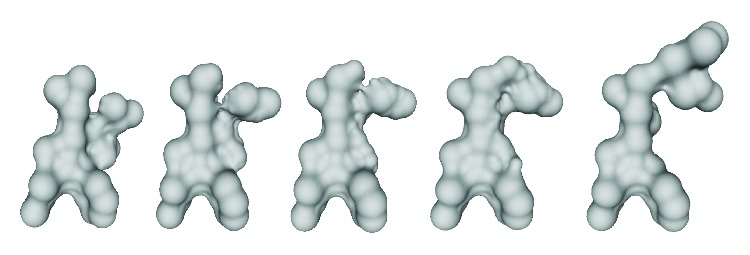
An example of partial molecular movement.

In real-world macromolecular dynamics, partial molecular movement simulation visualization is required, where the source and target shapes are from the same molecule in different forms. The molecule changes only part of its structure, and the rest of the atoms remain unchanged (Figure 7).

**Figure 8:**
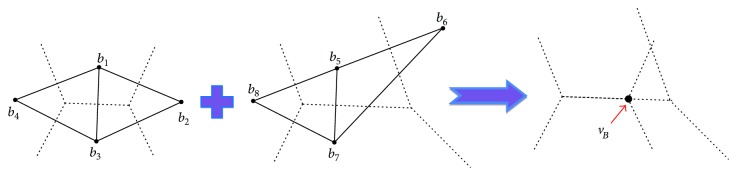
Superimposition of two Voronoi complexes constructed by two weighted point sets belonging to one “molecule” with partial movement. While this simple four-atom “molecule” only moves one of its atoms, the resulting intermediate Voronoi complex (right-most) has degenerate Voronoi cells such as the Voronoi vertex *v*_*B*_, which is an intersection at an endpoint of an edge.

However, the SIGP assumption is again unavoidably violated if partial molecular movement happens. In Figure 8, we show a simple “molecule” with four atom centers in ℝ^2^, and one of its atoms moves to a different position. In the intermediate Voronoi complex, two Voronoi edges from two Voronoi complexes intersect at an edge endpoint *v*_*B*_ that is not allowed by SIGP assumption in GMSSTF algorithm. The dual Delaunay cell of *v*_*B*_ is a trapezoid, which is new to GMSSTF algorithm. In this section, we attack this more complex degeneracy problem and introduce more types of intermediate Delaunay and Voronoi cells during the MSS transformation.

### 4.1. Degenerate Intermediate Voronoi Complexes

In partial molecular movement, we allow

(20) $$\begin{array}{c} \dim\left(v_{X}\;\cap \;v_{Y}\right)>\dim\left(v_{X}\right)+\dim\left(v_{Y}\right)-d, \end{array}$$

where *d* is the Euclidean space dimension.

In ℝ^3^, we have seven additional degenerate cases for Table 2, namely, (2,2,2), (2,1,1), (2,0,0), (1,1,1), (1,1,0), (1,0,0), and (0,0,0) (see Table 3).

**Figure 9:**
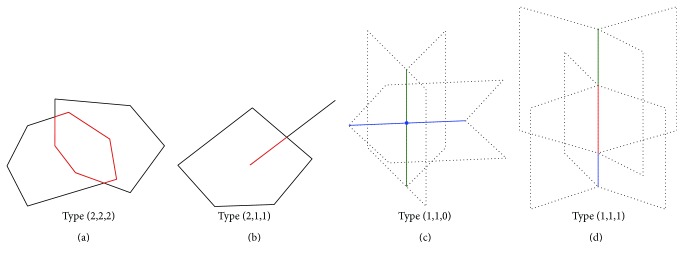
In (a), two Voronoi polygons overlap to form a new intermediate Voronoi polygon. In (b), a Voronoi polygon intersects a Voronoi edge and forms another Voronoi edge. In (c), two Voronoi edges intersect at an intermediate Voronoi vertex. In (d), two Voronoi edges overlap to form an intermediate Voronoi edge.

For each type, we demonstrate the shape of Voronoi cells and further conclude the shape of Delaunay cells using (14). In type (2,2,2), two coplanar Voronoi polygons overlap with each other, and the intersection is a polygon (Figure 9(a)). There are two Voronoi regions sharing this polygon. The two weighted points of these two regions form a convex hull that is a Delaunay edge. In type (2,1,1), a Voronoi edge intersects a Voronoi face and forms another Voronoi edge (Figure 9(b)). The resulting intermediate Voronoi edge is surrounded by four or five Voronoi regions, which are contributed by four or five intermediate weighted points. The convex hull of these weighted point centers is a quadrangle or pentagon. In type (1,1,0), two Voronoi edges intersect at a Voronoi vertex (Figure 9(c)). The Voronoi vertex is surrounded by nine Voronoi regions. The intermediate Delaunay cell is a 9-vertex polyhedron. In type (1,1,1), two Voronoi edges overlap and form an intermediate Voronoi edge (Figure 9(d)). There are minimally 3 and maximally 6 Voronoi regions surrounding this intermediate Voronoi edge. The intermediate Delaunay cell is a polygon with 3 to 6 vertices. For types (2,0,0), (1,0,0), and (0,0,0), the intermediate Voronoi cell is always a vertex. The intermediate Delaunay cells are obtained by counting the number of surrounding Voronoi regions.

Although the number of intermediate Voronoi cell types increases, the types of skin patches remain the same as hyperboloids and spheres. The transformation of intermediate skin surfaces still follows the approach described in Section 3.4.

## 5. Results

We apply the GMSSTF to both CPU and GPU approaches of MSS modeling. Suppose the source molecule consists of *m* atoms and the target molecule consists of *n* atoms. In the process of transformation, both CPU and GPU approaches reconstruct each intermediate MSS using *m* × *n* balls. It is noted that both approaches construct the underlying Voronoi and Delaunay complexes and mixed cells on CPU in the first phase (Figure 4). In contrast, the GMSSTF precomputes the fixed underlying Voronoi complex and transforms the Delaunay complex and mixed cells using linear interpolation. The new approach largely saves the time in the MSS transformation process.

Nine closely related molecule structures are downloaded from the Protein Data Bank (http://www.rcsb.org/pdb/) [4] to form ten different transformation processes. The PDB IDs of the nine molecules are 1J5F, 100D, 133D, 101D, 1AIE, 1D63, 114D, 161D, and 1XD7. All downloaded files are followed with extension ^∗^.pdb. In Table 4, we compare the CPU construction time with/without GMSSTF using Kruithof and Vegter's approach (KVA) [26] and Cheng and Shi's quality mesh approach (CSA) [28]. The experiment is performed on a machine with Intel i5@2.7 GHz and 8 GB RAM. Each transformation process generates 1000 intermediate MSSs. The average time taken for each intermediate MSS with the source and target molecules and the number of atoms is listed in Table 4.

**Figure 10:**
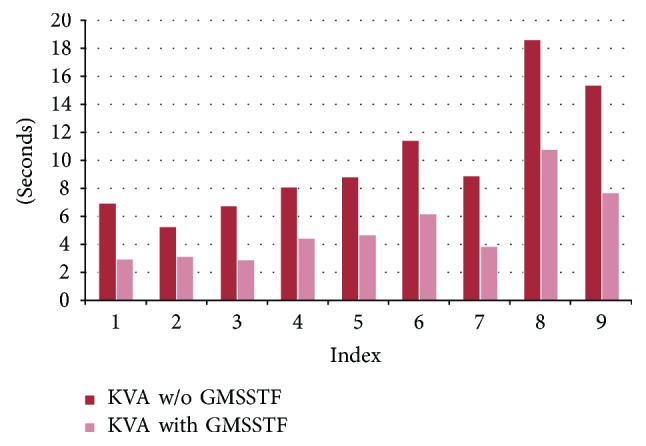
Computational speed comparison of using KVA with/without GMSSTF.

**Figure 11:**
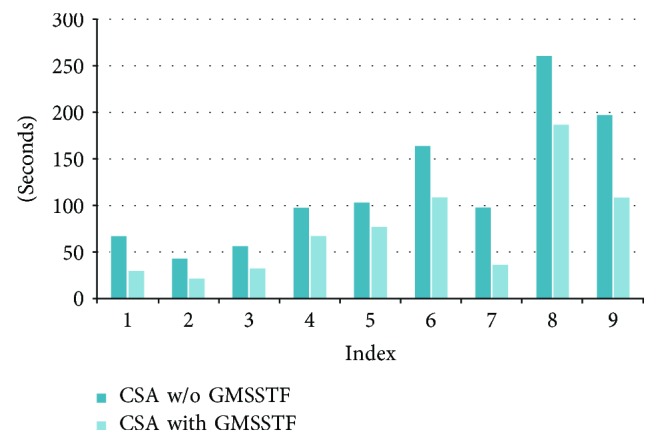
Computational speed comparison of using CSA with/without GMSSTF.

Figures 10 and 11 show the computational time differences using KVA and CSA with/without GMSSTF, respectively. It is clear that the GMSSTF largely reduces the computational time on generating intermediate MSS in the transformation process because of the reuse of intermediate Delaunay and Voronoi complexes. The average speedup is around 0.9.

**Figure 12:**
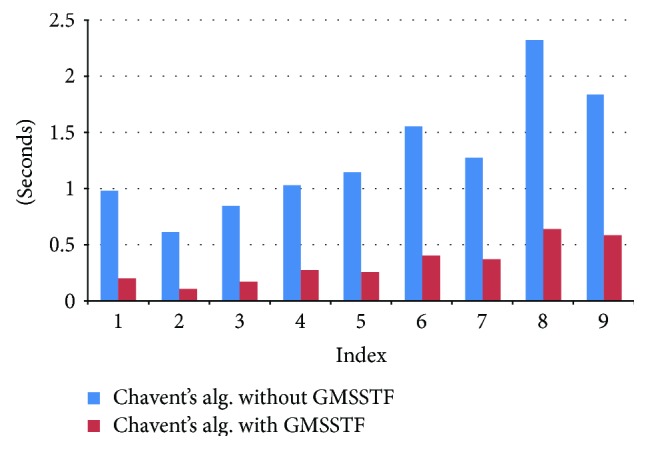
Computational speed comparison on GPU implementation based on Chavent's algorithm with/without GMSSTF.

We also implement the GPU visualization on MSS using Chavent's algorithm. It is noted that the first phase of Chavent's algorithm utilizes the same approach as KVA and CSA to generate the intermediate Voronoi and Delaunay complexes, as well as the mixed cell and MSS patch function. Therefore, the GMSSTF again effectively improves the performance on GPU implementation of MSS (Table 5 and Figure 12). The average speedup is around 3.2.

It is noted that both CPU and GPU approaches are able to generate high-quality transformation animations between macromolecules. Two supplementary videos available online at https://doi.org/10.1155/2017/4818604, namely, “114D-161D.wmv” and “161D-1DX7.wmv,” are attached to this paper to show the transformation from 114D to 161D and from 161D to 1DX7, respectively. The only difference exists only in terms of time complexities. For fare comparison purpose, we use many-to-many mapping between the source atoms and target atoms, which maps each atom from the source molecule to each atom from the target molecule. All produced animation processes are therefore identical. By using the proposed GMSSTF, we have shown the significant efficiency improvement for all approaches.

## 6. Conclusions and Discussions

In this study, we introduced a general MSS transformation framework which is potentially useful in mimicking biological macromolecular dynamics in bioinformatics research. We illustrated the solutions for a transformation process from one molecule to another as well as the transformation of a single molecule in different forms. The proposed framework requires no anchor point or skeleton information and handles topology changes automatically.

In order to validate the effectiveness of the proposed approach, we applied our method on the PDB database to model the biological macromolecular surface transformation process. The simulation results show that our method speeds up the traditional approaches. Although the traditional approaches, such as KVA, CSA, and Chavent's algorithm, also produce the similar animation process without GMSSTF, the construction of the first phase, that is, the Delaunay and Voronoi complexes and mixed cells, is recomputed for every intermediate frame. With GMSSTF, we reuse the previous frame's information to build the next frame's MSS with only translation and scaling operations. The new approach results in significant efficiency improvements using different PDB files.

In fact, each of the KVA, CSA, and Chavent's algorithm can generate different transformation processes if we assign atom correspondences manually (by consulting experts). However, for simplicity and fare comparison purposes, in this study, we use many-to-many mapping which assigns each atom from the source molecule corresponding to each atom from the target molecule.

As a future work, we will consult biologist for the usefulness of such an animation process. To mimic the real-world biological macromolecular dynamics, local control will be required, which is not considered in the current GMSSTF. Moreover, for given source and target molecules, different transformation processes will be generated for the biologist to choose. This can be done by employing the concept of shape space [44].

## Supplementary Material

A MSS transformation between a molecule with PDB indexed 114D and another molecule with PDB indexed 161D.
A MSS transformation between a molecule with PDB indexed 161D and another molecule with PDB indexed 1DX7.

## Figures and Tables

**Table 1:** Possible combinations of Voronoi cells, Delaunay cells, mixed cells, and skin patches.

Type	Voronoi cell	Delaunay cell	Mixed cell	Skin patch
3	Polyhedron	Vertex	Polyhedron	Sphere
2	Polygon	Edge	Prism	Hyperboloid
1	Edge	Triangle	Triangular prism	Hyperboloid
0	Vertex	Tetrahedron	Tetrahedron	Sphere

**Table 2:** Possible combinations of intermediate Voronoi cells, Delaunay cells, mixed cells, and skin patches.

Index	Type	Voronoi	Delaunay	Mixed cells	Patch
0	(3,3,3)	Polyhedron	Vertex	Polyhedron	Sphere
1	(3,2,2)	Polygon	Edge	Right prism	Hyperboloid
2	(3,1,1)	Edge	Triangle	Right Tri. prism	Hyperboloid
3	(3,0,0)	Vertex	Tetrahedron	Tetrahedron	Sphere
4	(2,2,1)	Edge	Parallelogram	Parall. prism	Hyperboloid
5	(2,1,0)	Vertex	Tri. prism	Sheared Tri. prism	Sphere

**Table 3:** Addition of types of intermediate Voronoi and Delaunay and mixed cells.

Type	Voronoi cells	Delaunay cells	Surface patch
(2,2,2)	Polygon	Edge	Hyperboloid
(2,1,1)	Edge	Quadrangle or pentagon	Hyperboloid
(1,1,1)	Edge	Polygon with 3 to 6 vertices	Hyperboloid
(1,1,0)	Vertex	Polyhedron with 9 vertices	Sphere
(2,0,0)	Vertex	Hexahedron or heptahedron	Sphere
(1,0,0)	Vertex	Polyhedron with 10 or 11 vertices	Sphere
(0,0,0)	Vertex	Polyhedron with 4 to 12 vertices	Sphere

**Table 4:** Performance of MSS modeling algorithms with and without GMSSTF. In general, GMSSTF saves half of the computational time on generating intermediate MSS.

Ind.	Source	Target	Number of atoms	KVA w/o GMSSTF (sec.)	KVA with GMSSTF (sec.)	Speedup (times)	CSA w/o GMSSTF (sec.)	CSA with GMSSTF (sec.)	Speedup (times)
1	1J5F	100D	174930	6.943 s	2.982 s	1.32	67.512 s	30.309 s	1.23
2	100D	133D	143080	5.253 s	3.137 s	0.67	43.835 s	21.613 s	1.03
3	133D	101D	162352	6.779 s	2.897 s	1.34	56.763 s	33.142 s	0.71
4	101D	1AIE	186260	8.124 s	4.431 s	0.83	98.021 s	67.647 s	0.45
5	1AIE	1D63	191955	8.819 s	4.710 s	0.87	103.812 s	77.572 s	0.34
6	1D63	114D	279624	11.446 s	6.203 s	0.85	163.972 s	109.653 s	0.50
7	114D	161D	198102	8.921 s	3.857 s	1.31	98.219 s	37.113 s	1.65
8	161D	1XD7	358498	18.635 s	10.824 s	0.72	259.912 s	187.653 s	0.39
9	1XD7	1J5F	315231	15.357 s	7.741 s	0.98	197.446 s	108.730 s	0.82

**Table 5:** Performance of MSS modeling algorithms on GPU (based on Chavent's algorithm) with and without GMSSTF. GMSSTF again effectively improves the performance on GPU implementation of MSS.

Ind.	Source	Target	Number of atoms	Chavent's alg. w/o GMSSTF (sec.)	Chavent's alg. with GMSSTF (sec.)	Speedup (times)
1	1J5F	100D	174930	0.985 s	0.203 s	3.85
2	100D	133D	143080	0.612 s	0.108 s	4.67
3	133D	101D	162352	0.846 s	0.172 s	3.92
4	101D	1AIE	186260	1.032 s	0.274 s	2.77
5	1AIE	1D63	191955	1.146 s	0.258 s	3.44
6	1D63	114D	279624	1.555 s	0.404 s	2.85
7	114D	161D	198102	1.273 s	0.372 s	2.42
8	161D	1XD7	358498	2.321 s	0.638 s	2.64
9	1XD7	1J5F	315231	1.836 s	0.584 s	2.14
